# Detection of Micro-Defects on Irregular Reflective Surfaces Based on Improved Faster R-CNN

**DOI:** 10.3390/s19225000

**Published:** 2019-11-16

**Authors:** Zhuangzhuang Zhou, Qinghua Lu, Zhifeng Wang, Haojie Huang

**Affiliations:** 1 Automation College, Foshan University, Foshan 528000, China; zhouzz_hn@126.com; 2 School of Mechatronics Engineering, Foshan University, Foshan 528000, China; zqhuanghaojie@163.com

**Keywords:** irregular surfaces, defect detection, K-Means, feature fusion

## Abstract

The detection of defects on irregular surfaces with specular reflection characteristics is an important part of the production process of sanitary equipment. Currently, defect detection algorithms for most irregular surfaces rely on the handcrafted extraction of shallow features, and the ability to recognize these defects is limited. To improve the detection accuracy of micro-defects on irregular surfaces in an industrial environment, we propose an improved Faster R-CNN model. Considering the variety of defect shapes and sizes, we selected the K-Means algorithm to generate the aspect ratio of the anchor box according to the size of the ground truth, and the feature matrices are fused with different receptive fields to improve the detection performance of the model. The experimental results show that the recognition accuracy of the improved model is 94.6% on a collected ceramic dataset. Compared with SVM (Support Vector Machine) and other deep learning-based models, the proposed model has better detection performance and robustness to illumination, which proves the practicability and effectiveness of the proposed method.

## 1. Introduction

The research objects of traditional defect detection have been mainly planar surfaces, regular spherical surfaces, and measured surfaces with diffuse reflection properties. In recent years, with the increasing requirements for intelligence in detection systems, several scholars have begun to study the detection of defects on irregular reflective surfaces. Up-to-date visual inspection is a difficult process in this area. Research objects with irregular surface features include ceramic surfaces, fine-ground metal surfaces, and solar panels. When photographs are taken, their bright physical properties cause local exposure and can be affected by disturbances in the surrounding environment. Additionally, the difficulty of detection is increased because the surface’s high reflectivity and its irregular shape are coupled to each other. With the aim to solve the problem of detecting defects on irregular surfaces, the research object used in this study was sanitary ceramic with an irregular surface and specular reflection shaped features.

Uncertainty factors in production, such as spraying processes, product transportation, and personnel operation, can cause cracks, breakages, peeling, and other appearance defects on the surface of the workpiece. These defects restrict the normal use of the product to a large extent [1,2,3,4,5]. Therefore, the development of a sanitary ceramic defect detection system for identifying and locating defects is very meaningful. However, the automatic detection of defects on irregular surfaces involves several challenges, such as the defect size being too small, the appearance of different defect types being very similar, and the contrast between the defect and the background being too low. The types of defects identified in this study were pinholes, hard cracks, cracks, spots, pits, and impurities on the surface of sanitary ceramics.

To solve the above problems, researchers have carried out defect detection on irregular surfaces by manually extracting the shallow layer of the research object and feeding the features to a conventional machine learning classifier, such as Support Vector Machine (SVM). Shanmugamani et al. [6] calculated a histogram, the gray-level co-occurrence matrix, and texture features in images, and the classification results of surface defects in gun barrels were obtained using an SVM classifier. However, the algorithm needs to extract multiple texture features, resulting in excessive time consumption. Saeed et al. [7] used a closed operation for the morphological processing of defect areas on tiles and classified the defect using the one-to-many support vector machine. Li et al. [8] located the defect profile of a track by using a trajectory extraction program and a projection algorithm. Feng et al. [9] proposed a probabilistic model for detecting the wear or loss of railway fasteners. The quality of a defect detection method based on traditional image processing depends largely on the quality of the extracted features. Therefore, when defect profiles are very similar, the recognition effect of the model is not optimal. In addition, when a new type of defect occurs, the feature extractor needs to be redesigned; thus, such algorithms are not robust to the defect shape.

In recent years, with the development of deep learning [10,11], target measurement algorithms based on Convolutional Neural Networks (CNNs) have achieved great success in many fields. A deep learning model avoids the cumbersome image processing steps of the traditional method by autonomously extracting high-dimensional information in the graph. Some researchers have tried to apply deep learning to the detection of defects on irregular surfaces. Maestro-Watson et al. [12] proposed a U-Net-based Fully Convolutional Neural Network (FCN) for the semantic segmentation of defects on reflective surfaces. The network performed a pixel-wise classification on the basis of local curvatures and data modulation to determine the location and boundaries of defects. Excellent recognition performance was achieved in industrial environments. Park et al. [3] detected defects such as dirt and scratches on the surface of a product by constructing deep networks with different depths and layer nodes. Zhao et al. [13] trained a backpropagation network and SVM classifier by extracting the area feature of the target. This method could effectively segment the defect area, but the shallow feature expression ability was limited, resulting in low recognition accuracy. Xu et al. [14] proposed an improved Faster R-CNN model that combines the characteristics of different layers and applies the Soft-NMS algorithm to detect defects in a tunnel. However, because of the excessive number of regional proposals considered by Soft-NMS, the model detection speed was not ideal. Although these improved methods of deep learning-based defect detection have advanced recognition performance, for micro-defects with different contours on a reflective surface, they have poor positioning accuracy and do not extract shallow features with local detail information.

In summary, there is a need for a model with improved robustness to the defect profile and a feature matrix that is able to distinguish defects. Thus, this study used Faster R-CNN with powerful feature expression and a structure that can be easily modified as the recognition framework.

However, since the image of a sanitary ceramic surface defect is quite different from the image of a natural scene, the direct use of the standard Faster R-CNN network encounters the following challenges: some defects (such as pinholes) in the image are 15 × 15 pixels, and the image size is 640 × 480 pixels; thus, the target area is too small relative to the entire picture. Furthermore, similar contour features (such as cracks and hard cracks) increase the difficulty of distinguishing defects. In order to solve these problems and thus increase the model’s ability to recognize micro-defects on irregular surfaces, we introduced the K-Means clustering algorithm and multi-scale feature combination to Faster R-CNN.

The structure of this paper is as follows: Section 2 introduces the method proposed in this paper, and Section 3 shows the experimental results. Finally, Section 4 summarizes this article.

## 2. Proposed Method

### 2.1. System Description

In the optical experimental platform, we used the industrial camera OPT-CM900-GM-04; the CMOS resolution is 4096×2160 pixels (the size of the pixel is 3.45 μm), and the focal length of the lens (OPT-C2514-10M) is 25 mm. The working distance between the lens and the research object was about 250 mm. A coaxial light source was used to provide illumination for the experimental platform. This type of light source can effectively overcome the reflection problem of irregular surfaces and reduce the influence of reflection on the recognition. A light source controller was used to control the light intensity to expose the defect to the camera. The captured image format was JPEG, with 640×480 pixels, and the height of each pixel in the image was approximately 0.0345 mm (74.52 mm/2160 pixels = 0.0345 mm/pixel).

### 2.2. The Proposed Neural Network Structure

The task for target detection technology can be roughly divided into three phases: candidate region generation, feature extraction, and target classification [15]. Currently, most detection models use the sliding window technique [16,17,18] to generate candidate frames. R-CNN [19] first uses a selective search algorithm [20] to generate candidate regions and then extracts the features of each region, which are classified by SVM. Fast R-CNN [21] proposes a multi-target network that combines target classification and position regression into one network, but the initial frame creation is time-consuming. Faster R-CNN [22] uses a Region Proposal Network (RPN) to generate candidate boxes and then extracts the feature matrix for target classification and bounding-box regression. Compared with a detection model based on sliding window technology, Faster R-CNN has a more expressive feature map and faster detection speed.

**Figure 1:**
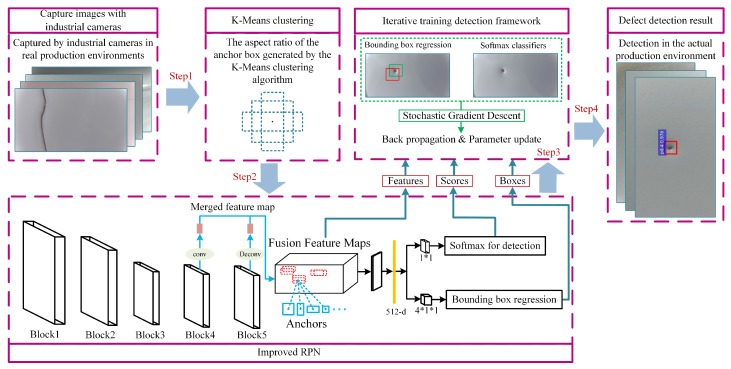
The proposed detection framework.

The feature extraction network of the Region Proposal Network has two types: the ZF [23] network and the VGG [24] network. The feature extraction network, determined by the characteristics of the research object in this study, needs to fully consider the global and local features of the images captured by industrial cameras. Therefore, in this study, the VGG network with a deep network layer and large modification space was selected as the feature extraction network. Our proposed detection framework is shown in Figure 1.

The innovation point mainly consists of two parts: (1) the K-Means clustering algorithm is used to generate the aspect ratio of anchor boxes, and the target area of all similar defects is marked by the bounding box that conforms to the morphological feature of the defect; (2) a multi-scale feature fusion network is used to extract the feature matrix in the prediction frame for subsequent defect identification and localization. The newly generated feature matrix combines the detailed features of the shallow network with the abstract features of the deep network. Compared with the standard network, it is more suitable for detecting micro-small targets; it has a higher positioning accuracy and a lower false detection rate.

#### 2.2.1. The K-Means Clustering Algorithm

The K-Means algorithm uses Euclidean distance as the classification standard and divides the data into several categories through unsupervised learning methods, which is consistent with the clustering requirements of the defects in this study. In this work, the number of cluster center points $k_{out}$ is determined by BWP(Between-Within Proportion) [25], which is the degree of intraclass tightness and the interclass dispersion of the samples. The aspect ratio of the defect sample is represented by *X*{*x*_1, *x*_2,…,*x*_*n*}, and the calculation method of $k_{out}$ is shown in Equation (1).

(1) $$k_{out}=max\left\{avgBWP\left(k\right)\right\},k\in \left[2,n\right)$$

The algorithm traverses the number of cluster center points in the range of [2, *n*). When *k* brings avgBWP(k) (defined in Equation (2)) to the maximum value, *k* is the number of cluster center points.

(2) $$avgBWP\left(k\right)=\frac{1}{n}\sum_{j=1}^{k}\sum_{j=1}^{n_{j}}BWP\left(j,i\right)$$

In the previous equation, BWP(j,i) is an evaluation index of the degree of tightness within the class and dispersion between classes. The calculation formula is shown in Equation (3).

(3) $$BWP\left(j,i\right)=\frac{b(j,i)-w(j,i)}{b(j,i)+w(j,i)}$$

In Equation (3), b(j,i) (calculated according to Equation (4)) represents the average distance from the *i*th point in the *j*th class to the samples in the remaining classes; w(j,i) (calculated according to Equation (5)) represents the average distance from the *i*th sample in the *j*th class to the rest of the samples in the class.

(4) $$b\left(j,i\right)=min\left(\frac{1}{n_{m}}\sum_{b=1}^{n_{m}}{\|x_{b}^{\left(m\right)}-x_{i}^{\left(j\right)}\|}^{2}\right),1\leq m\leq k,m\neq k$$

where *j, m* represents the category; *i* represents the number of the sample in the current category; $n_{m}$ represents the number of samples in class *m*; $x_{i}^{\left(j\right)}$ represents the *i*th sample in class *j*; $x_{b}^{\left(m\right)}$ represents the *b*th sample in class *m*; ${\left\|*\right\|}^{2}$ represents the square of the Euclidean distance.

(5) $$w\left(j,i\right)=\frac{1}{n_{j}-1}\sum_{q=1,q\neq i}^{n_{j}}{\left\|x_{q}^{\left(j\right)}-x_{i}^{j()}\right\|}^{2}$$

In Equation (5), $n_{j}$ represents the number of samples in class *j*, and $x_{q}^{\left(j\right)}$ represents the *q*th sample in the *j*th class. From the above calculation, the value of the cluster center point $k_{out}$ and the new aspect ratio of the anchor boxes can be obtained.

**Figure 2:**
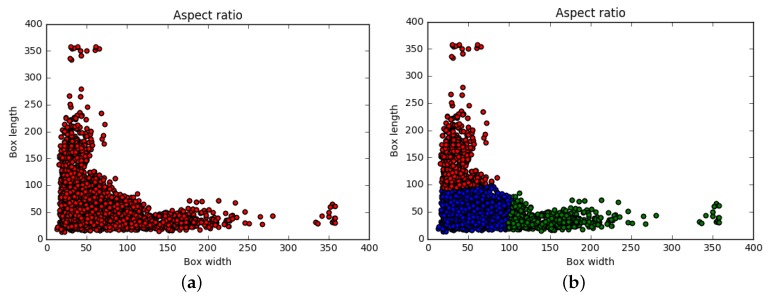
Aspect ratio. (**a**) Original aspect ratio, (**b**) aspect ratio after K-Means clustering.

An example of the distribution of the aspect ratio at the defect after clustering is shown in Figure 2.

In the figure, most of the defects are concentrated between (100, 100), (200, 50), and (50, 200). The number of cluster center points generated by the K-Means algorithm based on the BWP evaluation standard is 3.

After adjusting the aspect ratio of the anchor box, the base scale is further changed according to the morphological characteristics of the research object. The size of the anchor box in Faster R-CNN is $\left\{128^{2},256^{2},512^{2}\right\}$. Given the proportion of the defect in the background image, the initial anchor box with a size of 512 × 512 is too redundant, which wastes computing power. When the program refines the position of the initial bounding box, a large offset will occur, resulting in inaccurate positioning. This problem was addressed by adjusting the basic size of the anchor box to $\left\{64^{2},128^{2},256^{2}\right\}$ to improve the detection accuracy.

**Figure 3:**
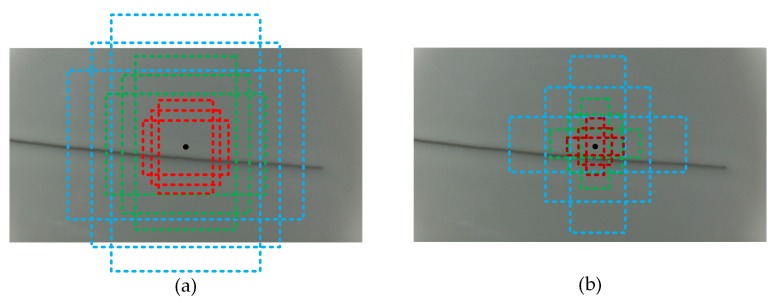
Anchor box comparison. (**a**) Original anchor box, (**b**) anchor box after K-Means clustering.

Standard Faster R-CNN and the partial initial anchor box based on the K-Means clustering algorithm are shown in Figure 3.

In Figure 3a, most of the candidate boxes contain too much background, which increases the computational cost of the algorithm and the difficulty of model convergence. In Figure 3b, most of the anchor boxes mark the location of the defect. The smaller candidate frame is useful for identifying pinholes, pits, spots, and impurity defects. The anchor boxes close to the rectangle are useful for identifying crack and hard crack defects; through a combination of various sizes and aspect ratios, the region proposal can cover most types of defects and effectively reduce the complexity of defect detection algorithms.

#### 2.2.2. Multi-Scale Fusion Feature Map

Ghodrati [26] pointed out that deeper networks can achieve higher recall rates, but the positioning accuracy is not optimal; shallow networks can obtain more detailed features with higher positioning accuracy but a lower recall rate. Since defects such as cracks and pinholes are small in size and irregular in shape, if the standard Faster R-CNN is used directly, misalignment and leak detection may occur. As a solution to this problem, a detection model for multi-scale feature fusion is proposed in this paper.

The quality of information contained in the feature map largely determines the final results of the network. As a deep network, VGG16 extracts features with a high degree of abstraction. However, the research object is micro-scale surface defects. Directly inputting the features extracted by VGG16 to the RPN will lead to inaccurate model positioning and low recognition accuracy. Therefore, in our approach, combinations of feature maps with different receptive fields are used as the input to the RPN layer to fully consider the global and local features of the defect and improve the detection performance of the model.

**Figure 4:**
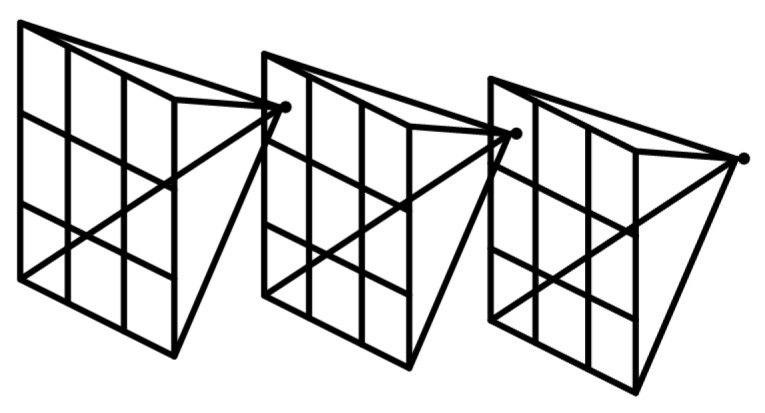
Receptive field plot.

The receptive field in deep learning (see Figure 4) refers to the mapping area of each pixel of the feature image of the original image.

The larger the neuron receptive field, the larger the range of original images that can be accessed: that is, the more abstract the features contained in the neuron, the higher the semantic level; the smaller the receptive field, the greater the tendency of the features in the receptive field to be local and detailed. The receptive field of a convolution in neural networks depends on the size of all the convolution kernels in the layer and the corresponding step size. The formula is shown in Equation (6):

(6) $$l_{k}=l_{k-1}+\left[\left(f_{k}-1\right)\times \prod_{i=1}^{k-1}s_{i}\right]$$

where $l_{k}$ is the receptive field of the *k*th layer of neurons, $l_{k-1}$ is the receptive field of the (k−1)th layer of neurons, $f_{k}$ is the size of the *k*th layer convolution kernel, and $s_{i}$ is the convolution step of the *i*th layer. From the above equation, the receptive field of the partial convolution layer of the feature extraction network VGG16 is obtained (see Table 1).

**Figure 5:**
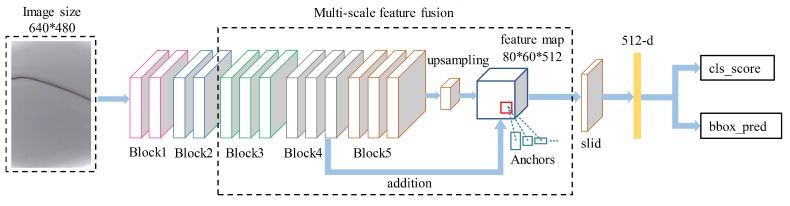
The multi-scale feature fusion framework.

In Faster R-CNN, the feature map received by the RPN layer is the output matrix of Conv5_3, and the receptive field in the original image is 196. This feature can better represent the contour information of the defect and obtain more accurate positioning results. However, for micro-sized defects with small differences between classes, the detection effect of this feature is not ideal because the expression ability of the feature with a high degree of abstraction is too low for micro-miniature defects. The problem of low feature expression is addressed in our approach by combining the outputs of the last two layers of the VGG network so that the newly generated feature map contains both global and local features of the defect. The multi-scale feature fusion network structure is shown in Figure 5.

**Figure 6:**
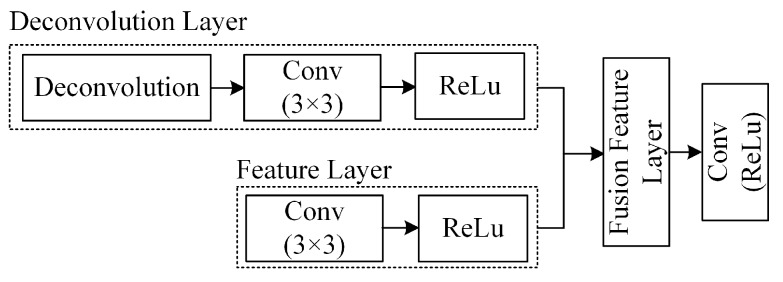
Deconvolution and the feature fusion structure.

In the multi-scale feature fusion network proposed in this paper, Block n represents the convolutional layer Conv n in the VGG16 network, and it has the same parameters as Conv n. The model input image size is 640 × 480 pixels. The multi-scale fusion feature map is composed of the output matrix of Block4 and Block5. The output of Block5 is upsampled, and its resolution is extended to 80 × 60. The detection framework upsamples the feature matrix by a deconvolution operation, as shown in Figure 6.

The model first expands the resolution of the input features by deconvolution, and then it convolves the layer features using a sliding window with a size of 3×3 and a step size of 1 to eliminate redundant data after deconvolution. After the feature is merged, the 3×3 convolution kernel is used again to eliminate the aliasing effect of the feature matrix.

The feature fusion of the detection framework is synchronized with the deconvolution. By combining the high-level features and the low-level features, the final feature map contains both semantic information and local features, thereby improving the ability of the feature map to express micro-miniature defects.

#### 2.2.3. Training the RPN

First, we trained the VGG16 network on the ImageNet dataset. Next, we used the trained network to initialize the convolution layer in the detection framework and then set a low learning rate to fine-tune the network. The network was iteratively trained 40k times, and we input a batch of marked data into the model during each iteration. When the candidate region generated by the RPN and the ground-truth box had an Intersection-over-Union (IoU) greater than 0.7, we marked it as foreground. When the candidate region and the IoU of all ground-truth boxes were less than 0.3, we marked it as background. The computational cost was reduced by selecting 256 candidate areas to participate in the training of the RPN, and the ratio of foreground to background was close to 1:1.

This system uses an end-to-end prediction method to integrate the bounding box regression and defect identification into a framework, and it optimizes the network parameters by using a multi-task loss function. The loss function is defined as follows, similar to Faster R-CNN:

(7) $$L\left(p_{i},t_{i}\right)=\frac{1}{N_{cls}}\sum_{i}L_{cls}\left(p_{i},p_{i}^{*}\right)+\lambda \frac{1}{N_{reg}}p_{i}^{*}L_{reg}\left(t_{i},t_{i}^{*}\right)$$

where *i* is the index of the candidate box, and $p_{i}$ is the probability that the candidate box is predicted to be foreground. When the candidate box is predicted to be foreground, $p_{i}$ is 1; otherwise, the value is 0. This means that the regression loss function is only activated if the candidate box is foreground. $N_{cls}$ and $N_{reg}$ are used to normalize the loss function, and λ is the balance parameter. $t_{i}=\left\{t_{x},t_{y},t_{w},t_{h}\right\}$ is a parameterized vector for the candidate region, and $t_{i}^{*}$ represents the coordinate vector of the real target frame.

After the RPN training was completed, the obtained candidate frame was repaired by using the obtained coordinate offset. Since the overlap of most candidate regions was severe, the non-maximum suppression algorithm was used to reduce the redundancy by eliminating redundant candidate regions according to the foreground confidence of the candidate frame. Finally, 2k proposals were selected for follow-up training. The bounding box loss function $L_{reg}$ of the multi-tasking loss function, as shown in Equation (8), is similar to Faster R-CNN.

(8) $$L_{reg}\left(t_{i},t_{i}^{*}\right)=\sum_{i}smooth_{L1}\left(t_{i}-t_{i}^{*}\right)$$

In the above formula, $t_{i}$ is the coordinate of the prediction frame represented by the vector, and $t_{i}^{*}$ is the ground-truth coordinate. The $smooth_{L1}$ loss function is shown in Equation (9):

(9) $$smooth_{L1}\left(x\right)=\left\{\begin{array}{c} 0.5\times x^{2},\;|x|<1\\ |x|-0.5,\;otherwise \end{array}\right.$$

Finally, the Softmax classifier was used to classify the defect targets of the candidate regions, and the relative probability values of each object belonging to category *i* were obtained, as shown in Formula (10):

(10) $$S_{i}=\frac{e^{V_{i}}}{\sum_{i}^{C}e^{V_{i}}}$$

where $V_{i}$ is the output of the upper level of the classifier, *i* is the index of the category, and *C* is the total number of categories. Its output is mapped between (0, 1), and the label corresponding to the maximum probability is the category of the predicted sample.

### 2.3. Dataset Description

**Figure 7:**
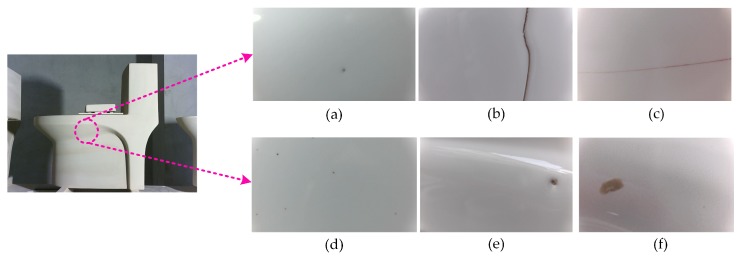
Defective sanitary ceramics. (**a**) Pinhole, (**b**) hard crack, (**c**) crack, (**d**) spot, (**e**) pit, (**f**) impurity.

This study focuses on defects existing on the surface of sanitary ceramics. In the actual engineering environment, a series of defects are generated on the surface of sanitary ceramics as a result of uneven glaze, unbalanced heat, or impurities falling onto the surface. The types of defects that the system needs to identify are shown in Figure 7.

**Figure 8:**
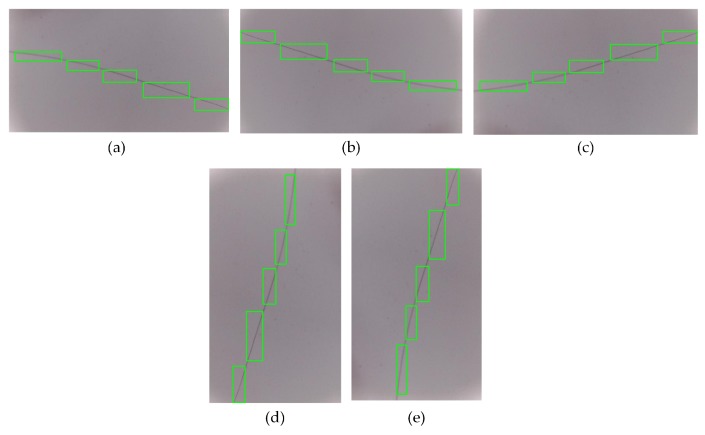
Data enhancement. (**a**) The original image, (**b**) rotated 180${}^{\circ }$ counterclockwise, (**c**) flipped horizontally, (**d**) rotated 90${}^{\circ }$ counterclockwise, (**e**) rotated 270${}^{\circ }$ counterclockwise.

The CNN model has a strong ability to express data, and it is prone to overfitting when the training set is small [27]; in this study, the sample collected by the experimental platform is limited, and the improved feature fusion network has a large depth. To avoid overfitting, we rotated and flipped the acquired samples to increase the diversity of the data. The data-enhanced sample set contains a total of 15,306 images, some of which are shown in Figure 8.

After using the above method to improve the diversity of the sample, the dataset was divided into three parts: the training set accounted for about 70%, the validation set accounted for about 15%, and the test set accounted for about 15%. It is worth noting that the training set and the test set were mutually exclusive; in other words, the test samples were not involved in the training process of the model. The division of labor of each sample set was as follows. The training set was input to the model in batch form during the training process to update the weight of the model; the validation set was used to select appropriate hyperparameters; and the test set was used to evaluate the detection performance of the model. The number of defect types included in each dataset is shown in Table 2.

Because of the small size and the irregular contour of the defects, excessive redundant information in the ground truth should be avoided when labeling the datasets. For micro-defects, such as pits, the label box should be the smallest circumscribed rectangle of the defect area. For slender defects, such as cracks, a segmented rectangular frame should be used to reduce the area of the non-defective area within the ground truth. The way in which a crack was marked is shown in Figure 8.

### 2.4. Detection Method

When the number of training samples is small and the differences between classes are not obvious, the model detection performance of the trained traditional method is weak. To efficiently train the defect detection framework of the profiled surface, we first increased the sample diversity by data enhancement and then generated the aspect ratio of the anchor box by the K-Means algorithm. The training process can be summarized in the following steps:

1. The defective sanitary equipment image is captured by an optical experimental platform.
2. The type and location of defects in the image are marked, and the diversity of the sample is increased by rotating and flipping transformations.
3. The K-Means algorithm is used to cluster the aspect ratio of ground truth. The new cluster center point replaces the aspect ratio of the anchor box in Faster R-CNN, and the base size of the anchor box is changed to make it more suitable for the research subject’s morphological characteristics.
4. The improved VGG16 network is used to extract multi-scale mixed features of the image. The extracted multi-scale feature map is used as an input to train the RPN to generate a series of region proposals.
5. Accurate regression of the previously generated region proposal is calculated by subsequent network and ROI pooling, and the defect types in the bounding box are identified by the Softmax classifier.
6. The area of the defect and its corresponding defect type are marked in the image.

## 3. Experimental Results

This section analyzes our proposed defect detection method. For the experiments, TensorFlow was selected as a deep learning framework. The program was run on an Intel core i7-8700 CPU, NVIDIA GTX-1070 GPU, 16 GB memory PC, and the operating system was Windows 10 1903. The test sample was 2295 images containing various types of surface defects.

### 3.1. Evaluation Indicators

To measure the detection performance of the proposed model, we used three common evaluation indicators, namely, Precision, Recall, and F-score. The precision measures the fraction of correctly identified positive detections and predicted positive detections, while the recall rate measures the fraction of correctly identified positive detections and true positive detections. The F-score is used to measure the overall performance of the model and is defined as follows:

(11) $$F-score=\frac{2\times Precision\times Recall}{Precision+Recall}$$

### 3.2. The Importance of K-Means

**Figure 9:**
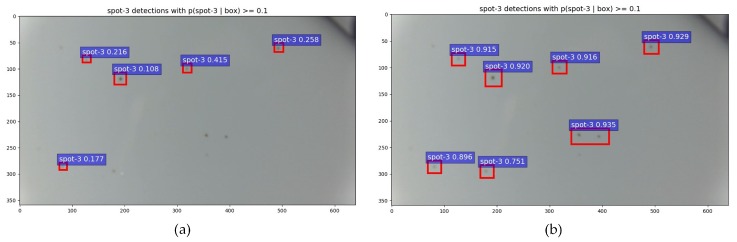
Comparison plot. (**a**) Defects detected by the standard network, (**b**) defects detected after adding K-Means.

Figure 9 shows the detection effect of Faster R-CNN on micro-miniature defects with and without the K-Means algorithm generating the aspect ratio of the anchor box. The figure shows that introducing the K-Means clustering algorithm increases the detection framework’s accuracy in locating small defects, such as spots. Additionally, the K-Means algorithm increases the confidence in the judgment of the defect type.

In the target detection task, an anchor box with a larger size contains more redundancy, which affects the precision of the model and increases the calculation cost. Therefore, we changed the base size n of the generated anchor box ($n\times 2^{3}$, $n\times 2^{4}$, and $n\times 2^{5}$) to 2, 4, and 8, respectively, and compared it with the standard network (whose base size is 16). By comparing the level of the F-score, we determined the most suitable size for the research object.

The model that participated in the detection of defects was trained in 40k iterations, and the confidence threshold was set to 0.5 in the test phase. The changes in the F-score at each stage of the model are shown in Table 3 at 10k iteration intervals. It can be seen from the table that the network with a base size of 8 always maintains good detection performance. However, the value of the base size is not as small as possible. When the base size is 2, the initial anchor boxes are too small to markedly weaken the detection performance of the model. Compared with the standard network (base size of 16), the model with a base size of 8 has the highest detection performance, and the F-score is increased by 0.015.

### 3.3. Analysis of the Multi-Scale Fusion Feature

After multi-scale feature fusion, the feature matrix can improve the recall rate of the defect detection model. To determine the best performance, we combined the features from different layers. These networks completed 40k iterations in training and had the same hyperparameters. Table 4 shows the recall rate and the F-score of the feature matrix composed of different layers.

The value 4-5 in the table indicates that the feature map is composed of the feature matrix output by Block4 and Block5, and Conv5_3 indicates that the feature map is only from the last layer output of the standard network. It can be seen from the table that when the feature matrix comes from Block4 and Block5, the model has the highest recall rate and F-score because the feature matrix combines abstract semantic information with local detail features. The recognition results of the feature fusion matrix from Block3, Block4, and Block5 are not ideal because the shallow features contained in Block3 have better positioning effects, but the discrimination of defects with similar contours is not optimal. The feature map from Conv5_3 in Faster R-CNN has a lower recall rate than that of 4-5, so we fused the features from Block4 and Block5.

**Figure 10:**
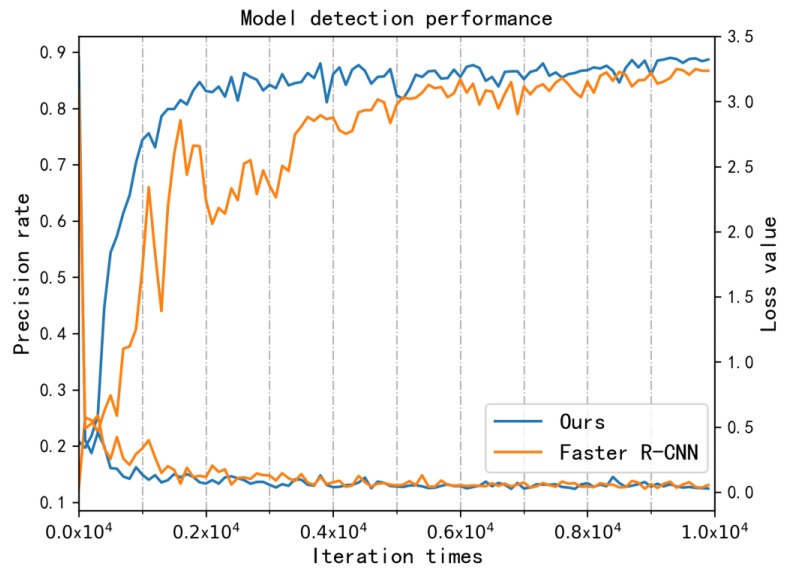
Comparison of the precision rate and loss function values between the two models.

To compare the convergence of Faster R-CNN with the improved model, we studied the change in the recognition precision rate and loss function values in the first 10k-iteration training cases. The models participating in the comparative experiment had the same hyperparameters: the learning rate was $10^{-3}$, the maximum number of iterations was $10^{4}$, and the weight decay rate was $5\times 10^{-4}$. The SGD (Stochastic Gradient Descent) algorithm was used as the optimizer with a test interval of 100 iterations. The detection performance line graph is shown in Figure 10.

In the figure, the loss values of the two models converge to around 0.1, indicating a good fitting effect. In the early stage of training, the precision of our algorithm has a faster ascending speed because the newly generated anchor boxes are more in line with the morphological characteristics of the research object, and the positioning accuracy of the micro-miniature defects is higher. For Faster R-CNN, the precision rate fluctuates greatly because the oversized initial anchor box produces more redundant information. After entering the steady rising phase, the improved model’s ability to perceive micro-miniature defects with similar contours is further enhanced, which increases the model’s recognition accuracy. In the first 10k iterations of training, the average precision of the improved model increases by 8.17%.

### 3.4. Comparison of Four Models for Micro-Defect Recognition

We compared the proposed method with other test frameworks. Table 5 shows the performance indicators of the detection frameworks of Faster R-CNN, YOLO-V3 [28], SVM + LBP (Local Binary Pattern) [29], and our method on the datasets we collected. The core idea of the YOLO (You Only Look Once) target detection framework is to use the global information of the entire image to directly calculate the position of the bounding box and its type using the regression method in the output layer, and it has a faster detection speed. SVM is a typical machine learning method that uses statistical concepts to solve classification problems. It extracts the texture features at the defects and maps them to high-dimensional space to complete the training and detection of the classifier. SVM is suitable for classifying small-scale data.

It can be seen from the table that the method we propose has the best detection performance. SVM has poor recognition performance for micro-defects with irregular contours because only the shallow features of the image are extracted. From the detection time, YOLO-V3 has a faster detection speed, but the precision and recall are too low, and the practicality is not ideal. Because the proposed algorithm upsamples the features of different layers and fuses the features, the detection time is longer. However, because a production line has a low running speed and requires high precision, the detection time still meets real-time requirements.

**Figure 11:**
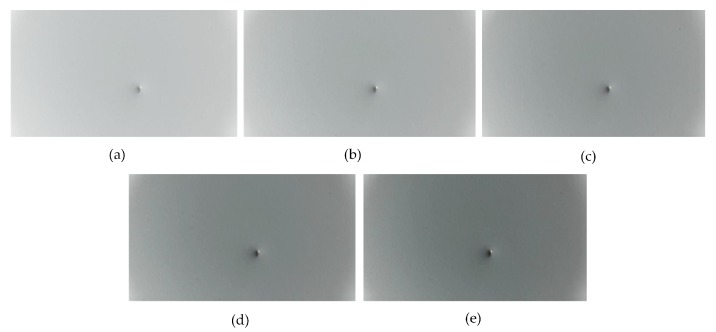
Defect samples with different contrasts. (**a**) Contrast parameter = 0.5, (**b**) contrast parameter = 0.75, (**c**) contrast parameter = 1.0, (**d**) contrast parameter = 1.25, (**e**) contrast parameter = 1.5.

In addition, to further verify the robustness of the proposed method in an actual application scenario, we simulated illumination changes by changing the contrast of the image in the test set and directly tested the performance of our model using these samples. The test samples under different contrasts are shown in Figure 11.

The F-scores under different lighting conditions are shown in Table 6.

It can be seen that the model has the highest F-score when the brightness of the test sample is similar to that of the training sample. When the brightness has a certain fluctuation, the detection performance of the model does not change significantly. These results prove our method’s robustness to illumination.

## 4. Conclusions

In this paper, we propose an improved Faster R-CNN model for detecting micro-defects on irregular surfaces with specular reflection properties. The model uses the K-Means clustering algorithm to generate the initial size of the anchor boxes that conform to the morphological characteristics of the research object. The feature maps are combined with different receptive fields for defect classification and position regression. Compared with other inspection frameworks, our method has a better ability to detect micro-miniature defects. The type of defect can still be accurately identified when the illumination changes, which makes the model proposed in this paper more convenient to transplant into practical industrial applications. Furthermore, our proposed algorithm can also effectively detect defects on other profiled surfaces, such as solar panels and glass bottles. In the next step, we will focus on reducing the scale of the feature extraction network while maintaining the detection performance to improve the detection efficiency of the model. 

## Figures and Tables

**Table 1:** Part of the VGG16 receptive field.

Layer	Kernel Size	Stride	Receptive Field
Conv 1_1	3×3	1	3
Conv 1_2	3×3	1	5
Pooling 1	2×2	2	6
⋯	⋯	⋯	⋯
Conv 5_3	3×3	1	196
Pooling 5	2×2	2	212

**Table 2:** Defect type statistics for each dataset.

Type of Surface Defect	Training Set	Validation Set	Test Set	Total
Pinhole	772	166	165	1103
Hard crack	2543	546	545	3634
Crack	2381	511	510	3402
Spot	2217	474	475	3166
Pit	1330	285	285	1900
Impurity	1470	316	315	2101

**Table 3:** Detection performance at each stage.

Base Size	10 k/iter	20 k/iter	30 k/iter	40 k/iter
2	0.306	0.381	0.161	0.202
4	0.933	0.911	0.931	0.92
8	0.932	0.934	0.935	0.943
16	0.897	0.918	0.924	0.928

**Table 4:** Recall rate from different combinations.

Layer	Recall	F-Score
4-5	95.0%	0.948
3-4-5	91.2%	0.917
Conv5_3	93.8%	0.937

**Table 5:** Performance comparison between different methods.

Method	Recall	Precision	F-Score	Time
SVM	41.6%	60.3%	0.492	0.63 s
YOLO-V3	45.6%	59.1%	0.515	0.089 s
Faster R-CNN	91.4%	89.7%	0.905	0.106 s
Our Method	95.0%	94.6%	0.948	0.118 s

**Table 6:** Detection performance after changing the image contrast.

Brightness	0.5	0.75	1.0	1.25	1.5
F-score	0.905	0.941	0.948	0.934	0.922
